# Post-traumatic olfactory dysfunction: a scoping review of assessment and rehabilitation approaches

**DOI:** 10.3389/fneur.2023.1193406

**Published:** 2023-07-13

**Authors:** Rosaria De Luca, Mirjam Bonanno, Carmela Rifici, Angelo Quartarone, Rocco Salvatore Calabrò

**Affiliations:** IRCCS Centro Neurolesi “Bonino-Pulejo”, Messina, Italy

**Keywords:** post-traumatic olfactory dysfunction, neurorehabilitation, traumatic brain injury, olfactory training, olfactory assessment

## Abstract

Post-traumatic Olfactory Dysfunction (PTOD) consists of a complete or partial loss of olfactory function that may occur after a traumatic brain injury (TBI). PTOD may be linked to some neuropsychiatric features, such as social, cognitive and executive dysfunction, as well as behavioral symptoms, especially when TBI involves the orbito-frontal cortex. The diagnosis of PTOD is based on medical history and clinical data and it is supported by psychometric tests (i.e., subjective tools) as well as electrophysiological and neuroimaging measures (i.e., objective methods). The assessment methods allow monitoring the changes in olfactory function over time and help to establish the right therapeutic and rehabilitative approach. In this context, the use of the olfactory training (OT), which is a non-pharmacological and non-invasive treatment option, could promote olfactory function through top-down (central) and bottom-up (peripheral) processes. To better manage patients with TBI, PTOD should be detected early and properly treated using the various therapeutic rehabilitative possibilities, both conventional and advanced, also taking into consideration the emerging neuromodulation approach.

## 1. Introduction

Traumatic brain injury (TBI) is a leading cause of significant public health problems, since it often causes high disability and mortality rates. Indeed, TBI is often associated with physical and sensory disturbances, including not only the well-known auditory and visual disorders, but also olfactory problems, which are instead poorly investigated (1). Post-traumatic Olfactory Dysfunction (PTOD) is commonly described as the complete or partial loss of olfactory function due to the block of nasal nerve passages, olfactory nerve injury or concussions or hemorrhages in the olfactory centers of the brain (2). Prognosis of olfactory dysfunction depends on the etiologies because conductive smell loss shows a good prognosis after intervention compared to sensory-neural type. Bratt et al. (3) found that 8% of patients with moderate or severe TBI had anosmia, and 14% had olfactory dysfunctions lasting years after the trauma. The likelihood of PTOD has been linked to both the severity of injury and length of post-traumatic amnesia (4). PTOD can occur in 0–16% of patients with mild TBI, 15–19% of those with moderate TBI, and 25–30% of those with severe TBI (5), especially in terms of the perception of smell changes, It has been suggested that the site of injury could be strictly related to olfactory dysfunction. In fact, results from morphological magnetic resonance image (MRI) studies in PTOD patients pointed out that brain lesions were localized in the orbito-frontal, olfactory frontal cortex, and temporal lobes (6). Generally, PTOD is linked to some neuropsychiatric disorders including anxiety, depression, and impulsivity, especially when TBI occurs in the orbito-frontal (ventral prefrontal) areas (7, 8). In this way, PTOD leads to lower quality of life (QoL) compared to TBI patients without olfactory dysfunction, also because of the fear of exposure to hazardous substances (9, 10).

Indeed neuroanatomical and kinetic factors make the peripheral and central olfactory structures more susceptible to damage when TBI occurs, as reflected by the high prevalence (11) of PTOD and the related psychological and cognitive – behavioral symptoms. Traumatic facial and brain injury due to blast and incidents are common causes of smell alterations, including total loss of function (anosmia), decreased sensitivity (hyposmia), alterations in odor quality (dysosmia/parosmia) and hallucination (phantosmia) (12). In some cases, brain damage limited to the primary olfactory areas leads to anosmia, while damage to the orbitofrontal cortex provokes olfactory discrimination and recognition deficits due to the multisensory integration role of this brain area (13). In a more specific way, anosmia can be considered as manifestation of frontal lobe damage, and it is correlated with alterations in verbal fluency abilities and executive functions (14, 15). Other authors (16) found that anosmia is strongly associated with depression symptoms in TBI patients, likely for the anatomical relationship between the two functions. The orbital frontal cortex plays a key role in both mood regulation and recognition and differentiation of odors (17). Moreover, hyposmia could be accompanied by socially disinhibited behavioral alteration, which is likely linked to orbital frontal cortex damage, as for the cognitive deficits (18). Moreover, Neumann et al. (19) reported that 56% of moderate to severe TBI presented dysosmia/parosmia in addition to difficulty in interpreting facial expressions and emotions.

These authors identified that cognitive-emotional networks, which are important for recognition and empathy, were also involved in central olfactory functions suggesting that this may be related to more complex social functions. It has been hypothesized that dysosmia/parosmia and depressive and anxiety symptoms are linked to persisting alterations of frontotemporal structures, such as the hippocampus and the orbitofrontal cortex (20, 21), as demonstrated by neuroimaging studies (22, 23). Phantosmia has been described as an olfactory disturbance in which individuals perceive an odor in the absence of a stimulus that may disappear, improve or worsen over time (24–26).

Various medical treatments have been tried to improve PTOD, including topical and systemic steroids, but well-controlled studies still lack (27).

Other drugs such as Gingko biloba and vitamin B have not proven to be effective to treat olfactory dysfunction (28).

Although there are limited therapeutic options for patients with PTOP, about 16.8 to 27% of patients may experience some degree of spontaneous recovery, which is mainly due to the high degree of neuroplasticity of the olfactory system (29, 30).

Natural smell recovery mostly occurs within 1 year after the traumatic event. However, the chances of improvement are reduced after 2 years from PTOD. Olfactory training might be a promising modality for the treatment of PTOD. In this context, different studies (31, 32) have indicated the effectiveness of olfactory training (OT), i.e., daily exposure to certain odors, thanks to the possibility of boosting neural plasticity of the olfactory system.

The diagnosis of smell disorders is suspected by medical history and supported by clinical data as well as by the results of psychophysiological, electrophysiological and neuroimaging measures. Among the validated psychophysical tests, the Sniffin’ Sticks Test (SST) is the most commonly and widely used tool. A more objective and quantitative measurement of sensory smell loss following TBI can be recorded though the Olfactory Event-Related Potentials (OERPs), which allows one to observe electrophysiologically the function of the olfactory system and its changes (33, 34).

In a forensic traumatic event, the clinical picture and severity of the person need to be determined with the use of objective criteria, although there are still limitations in objectively evaluating olfactory dysfunctions and state the relationship between the event and its cause. Performing both subjective and electrophysiological tests together to detect olfactory dysfunctions that occur after a forensic incident enable provide more reliable results in diagnosis and treatment (35). The OERPs method may provide objective data in the evaluation of post traumatic anosmia from the medicolegal perspective, to identify specific factors and the degree of smell loss (36).

The aim of this review is to investigate the main features of PTOD, focusing on the assessment through subjective and objective tools, emphasizing at the same time, the role of the main rehabilitative approaches to treat smell impairments following TBI.

## 2. Methods

### 2.1. Search strategy

The studies included in this review were identified by searching on PubMed, Scopus, Web of Science and Cochrane library, using the following keywords: “post-traumatic olfactory dysfunction” OR “olfactory dysfunction in traumatic brain injury” AND “post-traumatic olfactory dysfunctions and cognitive manifestations” AND “post-traumatic olfactory dysfunction diagnosis” OR “post-traumatic olfactory dysfunction assessment” AND “olfactory rehabilitative training in traumatic brain injury” OR “post-traumatic olfactory dysfunction rehabilitation.”

### 2.2. PICO evaluation

We defined the search terms using the PICO (population, intervention, comparison, outcome) model. We considered patients affected by PTOD as population; intervention included both assessment tools and rehabilitation approaches (conventional or not) for PTOD; comparison consisted in other kind of tool/medication used to assess/treat PTOD; the outcome measures considered were smell recovery, quality of life and any kind of improvement in olfactory function, including neuroplasticity.

### 2.3. Inclusion and exclusion criteria

The inclusion criteria were (i) patients affected by moderate to severe TBI with OD; (ii) randomized clinical trials (RCT), pilot studies and systematic reviews, case control and retrospective studies published between January 2012 and September 2022; (iii) English language; and (iv) papers published in a peer-reviewed journal. Exclusion criteria were (i) case reports and narrative reviews; (ii) studies describing other kinds of post-traumatic dysfunctions; (iii) studies involving children and adolescents affected by PTOD; (iv) other etiology of OD (i.e., vascular accidents, ischemic and/or hemorrhagic, neurodegenerative).

### 2.4. Literature selection

**Figure 1:**
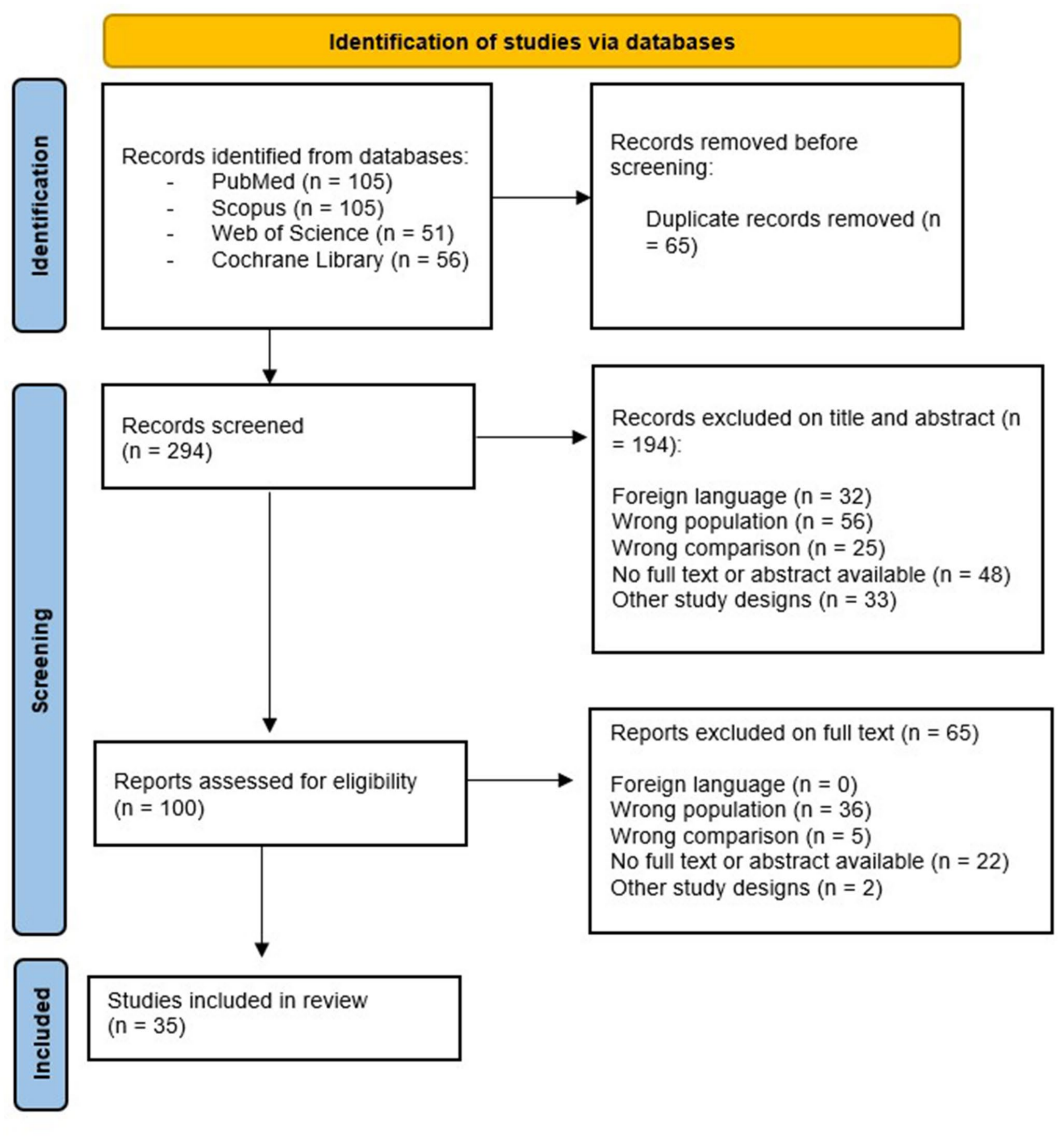
PRISMA flow chart for attrition of the papers, which were used in the final review.

Besides the papers themselves, we have analyzed the references of the selected articles, (but including only English papers), in order to obtain a complete search. The studies fulfilling our selected criteria and published between 2012 and 2022 were evaluated for possible inclusion (*n* = 198). Then, we have considered only English papers and removed duplicates (*n* = 100) (see Figure 1). Two reviewers (RDL and MB), have evaluated articles according to title, abstracts and text, and finally we considered 35 articles that addressed the main PTOD assessment tools and rehabilitative approaches.

### 2.5. Data extraction

In details, two reviewers (R.D.L. and M.B.) extracted data under the following categories: (i) measure characteristics (i.e., purpose, target population, time of test execution), (ii) psychometric properties of each assessment tool according to the information reported by the available studies and (iii) type of rehabilitative intervention used by the selected studies.

## 3. Subjective and objective assessment methods in olfaction

Recently, researchers made significant progress in the development of widely available, reliable, and reproducible methods to evaluate olfactory function. The administration of these tools is essential to establish the degree of chemosensory loss and confirm the patient’s complaint of olfactory alteration (37). Indeed, it permits monitoring the OD changes over time in post-TBI patients and helps to establish the right therapeutic and rehabilitative choice, considering also its impact on the patient’s treatment and counseling (38). Two main types of olfactory testing are commonly used: subjective tools, which include psychometric scales/tests (Table 1), and objective methods such as electrophysiological testing (Table 2) (62).

**Table 1:** The main subjective measures and complementary/additional tools to assess olfactory dysfunctions following TBI.

Subjective olfactory measures	Description
Screening tools	
Sniffin’ Sticks (SS)	Sniffin’ Sticks (SS) is a screening nasal test to evaluate smell function. It consists of a tool similar to a pen, which dispenses odors. It includes three tests: (i) for odor threshold (n-butanol, through one staircase), (ii) odor discrimination (through 16 pairs of smells, triple forced choice) and (iii) odor identification (16 common odorants, multiple forced choice from four verbal items per test odorant) (34). In particular, the SS presents a specific test–retest reliability for each subtest: *r* = 0.80 (odor discrimination), *r* = 0.88 (odor identification), and *r* = 0.92 (odor threshold). In addition, the SS’s sensitivity and specificity is about 84% of the total score (39, 40).
Brief Smell Identification Test (BSIT)	The Brief Smell Identification Test (BSIT) is an abbreviated version of the Smell Identification Test (SIT) used to measure olfactory function, especially in elderly population as possible clinical marker in Alzheimer disease (41). The BSIT is often used to screen olfactory function in elderly population. The execution takes about 5 min, including 12 different odors within scented strips and released when scratched with the tip of pencil. Each participant is submitted to a questionnaire with multiple-choice and asked to identify the odor corresponding to the odor strip for each smell. To complete the test, participants must indicate if there is any difficulty with smells (yes/no) to examine awareness. The BSIT is a forced-choice test, in which the patient is educated to identify each odor, even if no particular smell is perceived. This test has good internal reliability as well as validity. This is why the BSIT is a well-suited test for evaluating odor identification alterations in older people of different backgrounds, demonstrating a sensitivity of 63% and specificity of 88% with an overall accuracy of 71% (42).
Extensive, detailed testing of olfactory function	
University of Pennsylvania Smell Identification Test (UPSIT)	The UPSIT is used to assess the individual’s ability in detecting odors at a suprathreshold level, including 40 different smells. It consists in a quick self-administered test to quantitatively evaluate human olfaction (43). The UPSIT-40 has commonly been used in several countries as a diagnostic tool also in Parkinson Disease (PD) (44). Notably, UPSIT showed 82% sensitivity and 88.2% specificity (45, 46).
Barcelona Smell Test (BAST-24)	BAST-24 comprises 24 odors testing smell detection, identification, and forced choice. It is considered as a valid test to evaluate smell functioning [including the smell threshold, detection, memory, and identification (47)], in clinical practice. In addition, is can be useful to point out partial or total olfaction loss related to traumatic brain injury (48). It has been also validated in the Spanish and other Mediterranean populations.
Connecticut Chemosensory Clinical Research Center Test – (CCCRC)	The CCCRC test includes kits for odor detection and identification tests. The threshold evaluation is obtained using 9 serial dilutions of butanol in nanopore-deionized water. Each odor concentration is presented together with a control with water in a double-blind forced-choice paradigm. Given that, the threshold is described as the dilution at which the butanol bottle is correctly identified in 4 consecutive tests. If the water bottle is incorrectly indicated in less than 4 tests, the next higher concentration step is measured in a similar way (standard CCCRC test method). The CCCRC identification test comprises 7 smells (baby powder, chocolate, cinnamon, coffee, mothballs, peanut butter, and soap). Three smells stimuli (ammonia, Vicks and VapoRub) are administered to test trigeminal nerve nasal performance but are not included in the final score calculation. In fact, the test score is composed by a maximum of 100 points, adding the threshold score (a maximum of 50 points) and identification score (a maximum of 50 points) CCCRC olfactory test is considered one of the most reliable tests for assessment of olfactory function (49).
T&T olfactometry	The first standardized olfactometer in Japan was fabricated in 1975, and it included five test odors and the averages of the threshold concentrations. Nowadays, the T & T Olfactometer is a widely used tool not only in many clinics and laboratories but also in many prefectures and cities. Notably, the T&T reliability values were found in detection (*r* = 0.56) and recognition (*r* = 0.69), indicating a relatively accuracy in assessing patients’ olfactory ability (36, 48).
n-Butanol Threshold Test (n-BTt)	The n-BT allows the detection of the highest dilution of N-butanol correctly identified four times by the test subject. The patients are forced to choose between two options: odor or odorless. The identification test comprises 10 smells presented in pots and the subject is asked to pick them from a list of 20 proposals (50). This test is administered in clinical practice, to assess olfactory dysfunction in traumatic brain injury subjects
Olfactometer Test (O-Test)	Olfactometry is a precise testing method. Olfactory function can be tested through blast (Elsberg-Levy) olfactometry, a widely used tool to measure the olfactory threshold (34).T.his olfactometer determines the entry of a stream of air with a specific volume containing odorant molecules directly in the nasal cavity. The patient simultaneously presses the other nasal passage with his finger by pressing on the nostrils and shortly holding their breath. A clamp is located on the tube feeding air into the nasal cavity (51, 52).
Complementary/Additional measures	
Sino-Nasal Outcome Test (SNOT-22)	SNOT-22 is a questionnaire used to assess QoL in TBI patients. As the score increases, the QoL gets worse (Total score range: 0–110). SNOT- 22 may provide a valid instrument for the subjective QoL assessment of patients affected by PTOD (53). In particular, it has a sensitivity and specificity of 91.49 and 69.23%, respectively. Psychometric analyses support the accuracy, sensitivity, and specificity of the nasal domains of SNOT-22 to assess the impact on quality of life of the population with OD (54).
Diagnostic Assessment of Nonverbal Affect 2-Adult Faces (DANVA2-AF)	The Diagnostic Assessment of Nonverbal Affect 2–Adult Faces (DANVA2-AF) is often administered to evaluate facial affect recognition. Twenty-four faces are showed on computer screens and patients had to pick emotion (happy, sad, angry and fearful) from a list. Faces equally varied in sex, race, and expression intensity. Faces are showed on the screen for 15 s. Test scores range from 0 to 24 (55). The DANVA-2 presents some psychometric properties: internal consistency for adult and child faces subtests were 0.70 and 0.75, respectively. While test–retest reliability ranged from 0.78 to 0.84 (56).
Emotional Inference from Stories Test (EIST)	The Emotional Inference from Stories Test (EIST) consists in a set of 12 short tales, and it is administered to evaluate a participant’s ability to infer emotions from context. Each tale is presented one at a time on a computer with audio and video feedback. After that, patients had to answer a question about the character’s predominant emotion from a list of the following 4 options: happy, sad, angry, and fearful. They were not able to refer to the story to answer the question. Test scores range from 0 to 12. The EIST appears to be more sensitive to deficits in emotion inferencing abilities, as evidenced in the significantly lower scores on each version by people with TBI compared to healthy population (57).
Diagnostic Assessment of Nonverbal Affect 2-Adult Paralanguage (DANVA2-AP)	The Diagnostic Assessment of Nonverbal Affect 2-Adult Paralanguage (DANVA2-AP) is often administered to assess vocal affect recognition. One sentence that is neutral in content is presented orally 24 times with varying paralinguistic cues expressing different emotions. Participants should select the expressed emotion from a list of the following: happy, sad, angry, and fearful. The test scores range from 0 to 24. Notably, the test–retest reliability ranges from 0.73 to 0.93 (58).
Interpersonal Reactivity Index (IRI)	Empathy can be tested with the Interpersonal Reactivity Index (IRI), which tests total empathy and 4 empathy subtypes, using a self-report scale: perspective-taking (PT), empathic concern (EC), fantasy scale (FS), and personal distress (PD). The IRI has 28 items designed to capture these components of empathy. Participants had to estimate how well each statement described them through 5-point Likert scale. Total empathy scores range from 0 to 112. The psychometric properties of IRI include test–retest reliability and internal reliability (59). In adult population test–retest reliabilities of IRI ranged from 0.61 to 0.79 for males and 0.62 to 0.81 for females (60).

UPSIT, University of Pennsylvania Smell Identification Test; BAST-24, Barcelona Smell Test; SNOT-22, Sino-Nasal Outcome Test; VAS, Visual Analog Scale (0–100 mm); n-BTt, n-Butanol Threshold Test; SStt, Snap and Sniff Threshold Test; OTest, Olfactometric Test; SS, Sniffin’ Sticks; CCCRC, Connecticut Chemosensory Clinical Research Center Test; T&T, olfactometry; BSIT, Brief Smell Identification Test; DANVA2-AF, Diagnostic Assessment of Nonverbal Affect 2-Adult Faces; DANVA2-AP, Diagnostic Assessment of Nonverbal Affect 2-Adult Paralanguage; EIST, Emotional Inference from Stories Test; IRI, Interpersonal Reactivity Index.

**Table 2:** Description of the main objective measures to evaluate olfaction in patients affected by TBI.

Objective measures	Description
Electroencephalography (EEG)	Electroencephalography (EEG) in olfactory-bulb-associated areas can be used to assess the electrical activity changes in olfactory-bulb-associated brain regions (58). To investigate these areas high density EEG, using at least 128 channels, is fundamental.
Single Photon Emission Computed Tomography Image (SPECT-MRI) with Nasal Thallium-201	SPECT-MRI Image with Nasal Thallium-201 administration is a new assessment tool for olfactory nerve damage. This instrument is particularly useful to scan the olfactory nerve connectivity impairments in patients with olfactory alterations (61).
Event-related potentials (OERPs) Electrophysiological technique	An objective electrophysiological examination, using event-related potentials, to identify the olfactory defects (Elsberg Levy method – cortical evoked potentials) and to observe changes in olfactory functions (57).

EEG, Electroencephalography; SPECT-MRI Image with Nasal Thallium-201, Single Photon Emission Computed Tomography Image with Nasal Thallium-201; OERPs, Olfactory Event-related potentials.

Among the subjective examinations of olfaction, some screening tools are useful to differentiate easily and rapidly patients with normosmia, hyposmia or anosmia. In clinical practice, the most used screening tool for TBI-related olfactory dysfunctions is the Sniffin’ Sticks test, developed by Hummel in 1997, which contains some marker pens to be smelled, and it takes approximately 4 min to be administered. Concerning the recognition part, this validated test can be easily administered also in patients with language alterations (i.e., in the presence of aphasia) thanks to a wordlist of odors or non-verbal information like photographs/drawings representing smells. However, it requires good cooperation by the patient, who must pay attention during the test (39, 63).

When clinicians need to further investigate odor identification, discrimination and thresholds, a more extensive and detailed testing can be used (64) Indeed, the University of Pennsylvania Smell Identification Test (UPSIT), developed in the early 1980’s, focuses on the comparative ability of individuals to identify odors at the suprathreshold level (40–42).

It was administered also in Parkinson disease’s (PD) population and in patients with COVID-19 to reveal changes in the olfactory function (43–46).

Despite its high reliability (*r* = 0.94), the UPSIT has shown poor sensitivity for malingering detection in people familiar with the test mechanism.

The Connecticut Test, in which the odor stimulus is contained in suitable glass flasks (47) is quite similar to the Brief-Smell Identification Test (see Table 1), which is administered in older people and can be used for the evaluation and diagnosis of patients with olfactory impairments, considering the advantage of its low cost. An important issue about CCCRC is that low scores can be indicative of TBI, while abnormal detection thresholds may reflect altered olfactory cell function (49). Langdon et al. (48) evaluated severe smell loss in TBI patients using the Barcelona Smell Test (BAST-24), validated for the Catalan and Spanish population. It consists of 24 odors scoring smell detection, identification, and forced choice, and according to Cartesin et al., the tool is a good and reliable method to test the olfactory function in clinical practice (47).

Another specific test that can be administered to determine olfactory function in a rapid and non-invasive manner is the n-Butanol Threshold Test (n-BTt) (48, 65). Denzer et al. (50) used sniffing sticks with n-Butanol to investigate smell function, when generally this test is administered through gas chromatographic methods. The authors revealed that a pen set with n-butanol is an appropriate tool for testing olfactory sensitivity.

During the administration of self-assessment tools, there are three factors to consider: (i) odor threshold, (ii) odor discrimination, and (iii) odor identification (51, 52).In a recent study, Limphaibool et al. (2020) described a subjective olfactory examination, named the blast (Elsberg-Levy) olfactometry, which is a popular method of olfactory threshold measurement (34), in addition to the administration of main Fragrances Used in Olfactometer Test. The specific odors are mint (100% natural menthe piperita oil) and anise (100% natural *Illicium Verum* Seed Oil) at the temperature of 21 ± 1 degrees Celsius. Notably, anise oil is administered to stimulate the olfactory nerve endings whereas mint oil promotes the activation of both the olfactory and trigeminal nerve endings in the nasal mucosal tissue (66). Despite its usefulness in detecting olfactory thresholds, the blast (Elsberg-Levy) olfactometry may provide false results in smell performance due to the presence of odorant-free air, which could stimulate trigeminal nerve sensors (67).

Interestingly, Sattin (68) investigated olfactory function in patients affected by disorders of consciousness (DOC) due to TBI, using an olfactory discrimination protocol (ODP). This ODP was composed of four odors, selected and dosed according to both the literature on clinical sniff tests and to functional magnetic resonance, assessing the olfactory neural process and pathways (69–71). Given that the olfactory receptors are implicated in processing memory (which involves amygdala, hippocampus, etc.), olfaction could be a simple and direct way to stimulate memory and emotions in DOC.

Langdon et al. (48) administered the SNOT-22 as an additional/complementary outcome measure to investigate QoL in PTOD patients. In fact, the tool seems particularly useful in detecting changes in QoL according to smell symptoms (53, 54). In this vein, Neumann et al. (19) investigated the PTOD in moderate and severe TBI by assessing also emotional sequelae, through the administration of a complete battery, which included: (i) Olfaction (Brief Smell Identification Test-BSIT) (41); (ii) facial affect recognition [Diagnostic Assessment of Nonverbal Affect 2-Adult Faces (DANVA2-AF)] (55); (iii) vocal affect recognition [Diagnostic Assessment of Nonverbal Affect 2-Adult Paralanguage (DANVA2-AP)] (56); (iv) emotional inference [Emotional Inference from Stories Test (EIST)] (57) and (v) empathy [Interpersonal Reactivity Index (IRI)] (58). The authors showed that the detection of olfactory dysfunction may be related to affect and empathy deficits (59, 60). This is why an early assessment of these emotional impairments could be useful to set the most appropriate treatment for PTOD patients.

An objective examination (Table 2) may be indeed useful to more accurately identify olfactory defects.

Olfactory Event-related potentials (OERPs) are a reliable electrophysiological instrument to detect changes in olfactory function in an objective view. The recording device for cortical evoked potentials and odor stimulator, according to the above-mentioned Elsberg Levy method, can be also considered an objective olfactory evaluation (61). Compared to other methods such as MRI or fMRI, OERP measurements also have a higher temporal resolution, and can be conducted at lower cost with a lower degree of invasiveness (72).

Some authors have used OERPs to evaluate olfactory function in different patient populations such as multiple sclerosis (73) Alzheimer’s disease and other dementias (74–76), Parkinson’s disease (77), older people (78, 79), and as a marker for depression (80). However, other authors believe that these techniques are complex, time-consuming and not routinely performed in clinical practice (81). Notably, Shiga et al. used SPECT-MRI with Nasal Thallium-201 administration to identify lesions of the olfactory nerve connectivity in patients with impaired olfaction. In fact, these authors noticed that the degree of axon degeneration in human olfactory mucosa correlates with olfactory function (82).

## 4. Olfactory training: from the conventional to innovative approaches

**Figure 2:**
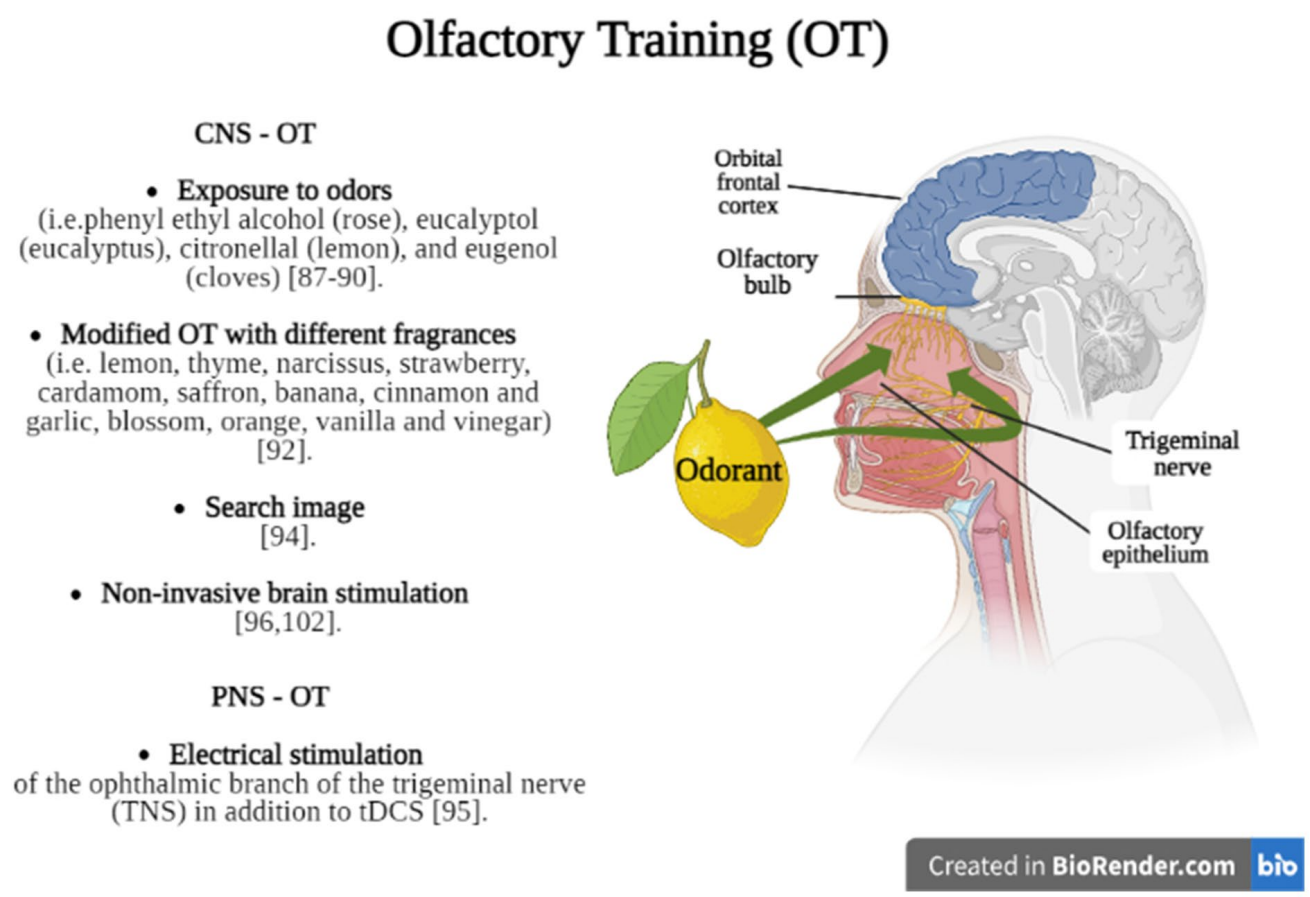
Briefly shows the main OT methods divided into techniques which stimulate olfactory function through CNS (CNS – OT) or PNS (PNS – OT) stimulation. Created in BioRender.com.

Olfactory training (OT) can be considered a non-pharmacological and non-invasive treatment option for patients affected by TBI with consequences in olfactory functioning (83), but also in individuals with signs of depression (84), neurodegenerative diseases such as Parkinson’s disease and older adults (85). Generally, OT consists in the administration of specific fragrances (i.e., floral, fruity, and more intense aromas such as eucalyptus) inhaled, which can stimulate the olfactory nerve and promote neuroplasticity (Figure 2).

Recently, a meta-analysis (86) found that OT was effective in 36.31% of PTOD patients who achieved clinically significant results after 8 months of training, while 27% of patients experienced spontaneous recovery of olfaction. In fact, OT could promote olfactory function through top-down (central) rather than bottom-up (peripheral) processes, as confirmed by Pellegrino et al. (87) and Konstantinidis et al. (88) applied to their patients a systematic OT for sixteen weeks, twice daily (in the morning and in the evening) and using four different odors, including phenyl ethyl alcohol (rose), eucalyptol (eucalyptus), citronellal (lemon), and eugenol (cloves); each odor was administered for ten seconds, with the same time interval of ten seconds between smells. They found that OT increased the identification and discrimination of olfactory functions in TBI patients, with positive effects also in cognitive functions. However, Jiang et al. (89) found that the administration of phenyl ethyl alcohol during OT produced improvements in olfactory thresholds in 23% of patients affected by post-traumatic anosmia but did not improve the odor identification ability. Using the Konstantinidis’s protocol (88), some authors suggested that the training is based on modulation of the regeneration processes linked to the repeated exposure to an odor, involving olfactory bulb and brain connectivity (90, 91).

Rezaeyan et al. (92), indeed, introduced a modified OT (Figure 1) stimulating the olfactory receptors with a variety of odorants over a certain period. The olfactory training was performed using four different packages including: (1) rose, lemon, thyme, and eucalyptus for the first month; (2) narcissus, strawberry, cardamom, and peppermint for the second month; (3) saffron, banana, cinnamon and garlic for the third month; (4) blossom, orange, vanilla and vinegar for the fourth month and led to positive results. It seems that the effectiveness of both OT, traditional and modified, depends on improved cognitive processing of olfactory information and increased attention paid to odors, supporting greater involvement of the CNS (93).

Laing et al. have described a rehabilitative method founded on the paradigm employed by Zelano et al., in which a neural representation of an odor is recalled from olfactory memory. In fact, the visual sighting of a food and imaging the food odor could activate the posterior piriform cortex that may allow a subject to perceive and identify the odor using central mechanisms only (94). Nevertheless, the effects of OT on neural structural changes due to the close connection between brain structure and olfactory function remains an unsolved question (95). For these reasons, more research is needed to clarify the optimal odor concentration, training duration, frequency, and the most suitable population for OT to better understand mechanisms underlying the recovery processes.

The olfactory nerve can also play a key role in such plastic and regenerative processes (96). Indeed, it has been shown that neurostimulation (about thirty minutes), delivered to the ophthalmic branch of the trigeminal nerve through trigeminal nerve stimulation and transcranial direct current stimulation, significantly improved the olfactory performance to guaiacol, an odorant involved in the activation of intranasal trigeminal circuit. In this way, both methods may induce persistent modulation changes through a direct activation of the trigeminal nerve. On the other hand, these neuromodulator effects may be driven *via* activation of distal secondary olfactory cortex structures, such as the orbitofrontal cortex which is highly associated with processing of odor learning and memory. In fact, non-invasive brain stimulation (NIBS) is considered an emerging and promising approach to induce long-lasting neuroplastic changes through electrical and/or magnetic energy. According to Hara et al. (97), the combined use of NIBS with rehabilitation could enhance a positive synergic effect, promoting not only modulation of neural connections, but also functional re-learning in post-TBI patients. However, the use of NIBS in the treatment of PTOD is an issue that deserves to be investigated.

## 5. Discussion

Olfactory dysfunction is an underestimated and challenging issue in TBI that worsens patients’ QoL, not only in eating and enjoyment of food, but also in hazard avoidance (gas leaks, smoke detection, chemical vapors, and rotten food). Our review suggests that PTOD is commonly associated with cognitive and neuropsychiatric sequelae in TBI patients due to OFC damage, and some authors reported that OD is also linked to the neurodegenerative pathology. Then, it could be considered as a clinical marker of neurodegeneration likewise other more direct clinical, biological and neuroimaging markers. For this reason, an early assessment of olfactive function should be implemented in clinical practice, especially when dealing with TBI, and this is why a comprehensive review on this issue is of utmost importance. In fact, it seems that olfactory testing, especially in the acute phase, could be useful as a screening tool for long-term outcomes, including mood symptoms. According to Logan et al., there is a bidirectional correlation between OD and depression, due to reduced input to the olfactory bulb and the consequential lower levels of neurotransmitter concentration, leading to the potential disturbance of emotional functioning (98).

PTOD is also related to other neuropsychiatric sequelae, including anxiety which affects odor thresholds, identification and discrimination of different smells. It seems that the amygdala and the OFC are both involved in anxious states and in the olfactory functioning (99). OD is also associated with impulsivity probably because OFC, is involved in both olfactory neural pathways and the regulation and inhibition of behavior (100).

Moreover, it has been shown that detection of olfactory abnormalities may be related to affect and empathy deficits (19), and this could be due to a common dopaminergic pathway dysfunction. Then, addressing OD and the related cognitive/behavioral dysfunction may be of help in better manage the rehabilitation of patients with TBI. Clinicians have a wide range of PTOD assessment methods, both subjective and objective, although it is not always easy to administer the right test or measure. The Sniffin’ Test, UPSIT and CCCRC are the most used in clinical practice for their rapidity and cost convenience. Despite their short duration, there are some limitations related to learning effects due to repeated testing, and the low resolution in terms of detecting changes. In detail, the CCCRC and the BAST-24 provide verbal odor identification which is strictly dependent on language function and cognition (101). This is why patients are exposed to a pre-selected list of odor descriptors without which there would be no reliable clinical results. However, the BAST-24 is particularly useful to detect not only olfactory changes, but also neurobehavioral disorders (i.e., eating) (100).

OERPs are instead more accurate to detect changes in olfactory function, despite their limited availability in standard health care and the high cost of administration. Nevertheless, objective examinations are particularly useful when level of cognition is too impaired or when subjects may exaggerate the smell deficit for a secondary gain or for other medico-legal reasons. In addition, OERPs allow to understand the site of damage: loss of smell without loss of OERPs suggest peripheral nerve lesions, while a reduction/absence of OERPs indicates damage to central olfactory system (72). Another important issue to consider is the best way to manage PTOD, although it is still an underestimated problem. Currently, evidence supports the use of topical corticosteroids that allow neuronal recovery following olfactory nerve transection through the reduction of the inflammatory reaction and decrease of glial scar formation (101). This may explain why corticosteroids combined with OT are more effective (102). Other medications to treat olfactory loss include supplementation with alpha-lipoic acid, vitamin A and omega-3 for their neurodegenerative potential and antioxidant properties. Other promising treatments are related to the administration of the experimental N-acetylcysteine (100 mg/kg twice daily) after acute olfactory neuronal injury in animal models, since it reduces neural loss in the olfactory bulb (103). In fact, the neuroprotective effect of this medication could provide clinical benefit also in the TBI population. OT has been introduced in patients’ care despite the lack of specific recommendations; moreover, its role in stimulating central or peripheral components of the olfactory system is mostly unknown. For this reason, the real effectiveness of OT remains a challenge, although it could be considered a good option to manage this growing and important problem. According to Turner et al. (104), a higher quality of evidence is needed with respect to patient populations, protocols, and outcome measures. Recently, researchers have studied the role of emerging approaches, including the use of NIBS that could boost neuroplasticity, further potentiating the OT after-effects (105–107). However, the lack of conclusive evidence does not allow to recommend this therapeutic approach in terms of efficacy. Another treatment for olfactory dysfunction is the use of platelet-rich-plasma which is derived from blood’s patient with pro-regenerative properties (108). Nevertheless, larger studies are needed to understand if it can be adaptable also in TBI patients. Finally, the traditional Chinese acupuncture (109), used for various medical conditions, was proven effective in post-viral infection patients who were refractory to other treatments, including OT, oral steroids and supplementation. Although the reporting information in this review followed the PRISMA guidelines to reduce bias, there are some limitations to acknowledge. Since we included only English papers, some studies may have been excluded based on the language criteria. In addition, we did not provide any statistical analysis for each study included because of our intention was to describe the most used tools and methods to assess olfactory function in PTOD and its rehabilitative approach, since an international consensus about a gold-standard does not exist yet.

## 6. Conclusion

In conclusion, an early assessment of olfactory sense, considering also its correlation with cognitive functioning, is recommended in clinical practice and especially in the rehabilitation of patients with TBI. Although no clear evidence exists on the best treatment option, OT could be considered a valuable and effective tool to promote neuroplastic processes and improve OD following TBI. Further research is needed to investigate the promising role of OT coupled to other emerging training methods in the management of patients with TBI and olfactory loss and/or alterations.

## Author contributions

MB, RC, and RL: conceptualization and investigation. MB and RL: methodology, data curation, and writing—original draft preparation. MB and CR: software. RL, MB, CR, AQ, and RC: validation and visualization. AQ: resources and funding acquisition. RC: writing—review and editing and supervision. All authors have read and agreed to the published version of the manuscript.

## Funding

This study was supported by Current Research funds 2023, Ministry of Health, Italy.

## Conflict of interest

The authors declare that the research was conducted in the absence of any commercial or financial relationships that could be construed as a potential conflict of interest.

## Publisher’s note

All claims expressed in this article are solely those of the authors and do not necessarily represent those of their affiliated organizations, or those of the publisher, the editors and the reviewers. Any product that may be evaluated in this article, or claim that may be made by its manufacturer, is not guaranteed or endorsed by the publisher.
